# Poster Session I - A75 TOTAL EXAMINATION TIME IN UPPER GASTROINTESTINAL ENDOSCOPY AND LESION DETECTION: A PROSPECTIVE STUDY

**DOI:** 10.1093/jcag/gwaf042.075

**Published:** 2026-02-13

**Authors:** S Mitchell, R Djinbachian, J Liu, E Bernard, R Battat, M Bouin, L D’aoust, D C Daoud, E deslandres, R Leduc, P Benoit, K Orlicka, S Bouchard, V Michal, D von Renteln

**Affiliations:** Centre de Recherche du Centre Hospitalier de l’Universite de Montreal, Montreal, QC, Canada; Centre Hospitalier de l’Universite de Montreal, Montreal, QC, Canada; Centre Hospitalier de l’Universite de Montreal, Montreal, QC, Canada; Centre Hospitalier de l’Universite de Montreal, Montreal, QC, Canada; Centre Hospitalier de l’Universite de Montreal, Montreal, QC, Canada; Centre Hospitalier de l’Universite de Montreal, Montreal, QC, Canada; Centre Hospitalier de l’Universite de Montreal, Montreal, QC, Canada; Centre Hospitalier de l’Universite de Montreal, Montreal, QC, Canada; Centre Hospitalier de l’Universite de Montreal, Montreal, QC, Canada; Centre Hospitalier de l’Universite de Montreal, Montreal, QC, Canada; Centre Hospitalier de l’Universite de Montreal, Montreal, QC, Canada; Centre Hospitalier de l’Universite de Montreal, Montreal, QC, Canada; Centre Hospitalier de l’Universite de Montreal, Montreal, QC, Canada; Centre de Recherche du Centre Hospitalier de l’Universite de Montreal, Montreal, QC, Canada; Centre de Recherche du Centre Hospitalier de l’Universite de Montreal, Montreal, QC, Canada

## Abstract

**Background:**

In colonoscopy, longer withdrawal time is an established quality indicator. In contrast, no equivalent benchmark exists for upper gastrointestinal endoscopy (EGD), despite evidence from Asian studies suggesting that longer examination time improves neoplasia detection.

**Aims:**

We aimed to evaluate the association between total EGD examination time and lesion detection in a large North American cohort.

**Methods:**

We performed a secondary analysis of a prospective study including diagnostic, surveillance, and screening EGDs performed at the Centre hospitalier de l’Université de Montréal (CHUM) between November 2023 and August 2025. Procedures with pre-planned polypectomies, interventions and known pathologies (e.g. Barrett’s esophagus) were excluded. Total examination time was measured from endoscope insertion to withdrawal. Detection rates for six abnormality categories (significant visual, significant biopsy/polyp, all significant, all visual, all biopsy/polyp, and all abnormalities) were calculated. Associations between procedure time and detection rates were modeled using logistic regression with generalized estimating equations to account for clustering by endoscopist.

**Results:**

Among 1169 included EGDs (mean age 55.9 years, 57.5% female), the mean total examination time was 5.6 ± 3.7 minutes. For the primary outcome, each additional minute of examination increased the odds of detecting at least one significant visual abnormality by 10.9% (p < 0.001). Similar associations were observed for all secondary outcomes, with odds ratios ranging from 1.13 to 1.25 per additional minute (all p < 0.001). Detection rates rose steadily until approximately 13–15 minutes, beyond which gains plateaued. These findings indicate that longer total EGD examinations correlate with higher lesion detection yield.

**Conclusions:**

Increasing EGD examination time yields higher detections of significant lesions. Minimum thresholds for total EGD examination time should become standard similar to colonoscopy withdrawal time recommendations.

**Funding Agencies:**

PREMIER, Faculty of Medicine, University of Montreal

